# Routinely asking patients about income in primary care: a mixed-methods study

**DOI:** 10.3399/BJGPO.2021.0090

**Published:** 2022-01-12

**Authors:** Andrew David Pinto, Erica Shenfeld, Tatiana Aratangy, Ri Wang, Rosane Nisenbaum, Aisha Lofters, Gary Bloch, Tara Kiran

**Affiliations:** 1 Upstream Lab, MAP/Centre for Urban Health Solutions, Li Ka Shing Knowledge Institute, St. Michael’s Hospital, Toronto, Canada; 2 Department of Family and Community Medicine, St. Michael’s Hospital, Toronto, Canada; 3 Department of Family and Community Medicine, Faculty of Medicine, University of Toronto, Toronto, Canada; 4 Division of Clinical Population Health, Dalla Lana School of Public Health, University of Toronto, Toronto, Canada; 5 Institute for Health Policy, Management and Evaluation, Dalla Lana School of Public Health, University of Toronto, Toronto, Canada; 6 MAP/Centre for Urban Health Solutions, Li Ka Shing Knowledge Institute, St. Michael’s Hospital, Toronto, Canada; 7 Division of Epidemiology, Dalla Lana School of Public Health, University of Toronto, Toronto, Canada; 8 Department of Family and Community Medicine, Women’s College Hospital, Toronto, Canada

**Keywords:** inequalities, social determinants of health, income, socioeconomic factors, surveys and questionnaires, primary health care

## Abstract

**Background:**

Income is a key social determinant of health, yet it is rare for data on income to be routinely collected and integrated with electronic health records.

**Aim:**

To examine response bias and evaluate patient perspectives of being asked about income in primary care.

**Design & setting:**

Mixed-methods study in a large, multi-site primary care organisation in Toronto, Canada, where patients are asked about income in a routinely administered sociodemographic survey.

**Method:**

Data were examined from the electronic health records of patients who answered at least one question on the survey between December 2013 and March 2016 (*n* = 14 247). The study compared those who responded to the income question with non-responders. Structured interviews with 27 patients were also conducted.

**Results:**

A total of 10 441 (73%) patients responded to both parts of the income question: *‘What was your total family income before taxes last year?’* and *‘How many people does your income support?’*. Female patients, ethnic minorities, caregivers of young children, and older people were less likely to respond. From interviews, many patients were comfortable answering the income question, particularly if they understood the connection between income and health, and believed the data would be used to improve care. Several patients found it difficult to estimate their income or felt the options did not reflect fluctuating financial circumstances.

**Conclusion:**

Many patients will provide data on income in the context of a survey in primary care, but accurately estimating income can be challenging. Future research should compare self-reported income to perceived financial strain. Data on income linked to health records can help identify health inequities and help target anti-poverty interventions.

## How this fits in

The relationship between income and health is well known but data on income is rarely collected. From 14 247 patients who completed at least one question on a routinely offered sociodemographic survey, 73% responded to both parts of the income question, and were most comfortable answering if they understood the income-health relationship and believed data would be used to improve care. Data on income can support the identification of health inequities to directly address social needs, including through emerging anti-poverty interventions.

## Introduction

Income has long been recognised as one of the most important social determinants of health, as it determines access to basic necessities and is a key part of social status.^1,2^ A large body of research demonstrates the strong connection between poverty and poor health outcomes.^3–5^ Even in countries with universal health insurance, income is often associated with access to health services, with poorer patients experiencing worse access to health services and worse outcomes.^6–8^

Routine collection of data on income is an important step to identifying inequities in access and outcomes. Clinicians may not be able to accurately identify which patients are living in poverty, particularly in mixed-income neighbourhoods.^9,10^ Other studies have found that physicians frequently overestimate patients’ income.^11,12^ Data on income can be used to pinpoint health inequities that could be amenable to quality improvement initiatives, such as focusing more time and attention to engage low-income patients, providing support to help these patients engage in care (for example, transportation vouchers), and considering issues such as food insecurity when developing care plans.^13,14^ Data on income can also be used to target clinic-based interventions to address financial strain as a key social determinant of health.^15,16^ A small but growing number of interventions exist to improve a patient’s income.^17^ In the UK, general practices have hosted charities that help patients access financial benefits.^18–21^ In one Canadian primary care organisation, patients can be referred to a specialised income security health promotion programme.^22^ Medical-Financial Partnerships that help patients access financial benefits and complete their taxes are beginning in several US cities.^23,24^

There is little research on how patients respond to being directly asked about their income. The authors’ primary care organisation has often asked about income in the context of a routinely administered sociodemographic survey.^25^ The objective was to evaluate non-response bias, compare answers provided with other data available on income, and examine how patients reacted to being asked about their income.

## Method

### Setting and context

In Canada, permanent residents have access to publicly funded health insurance that covers the cost of most medically necessary services. This study took place at the St Michael’s Family Health Team, a large primary care organisation with approximately 70 staff physicians, 40 resident physicians, and >60 other health professionals, serving approximately 45 000 patients at six clinics in downtown Toronto, Canada. The Family Health Team has a specific mandate to serve marginalised populations, but serves a diverse cross-section of the community.

**Figure 1.**
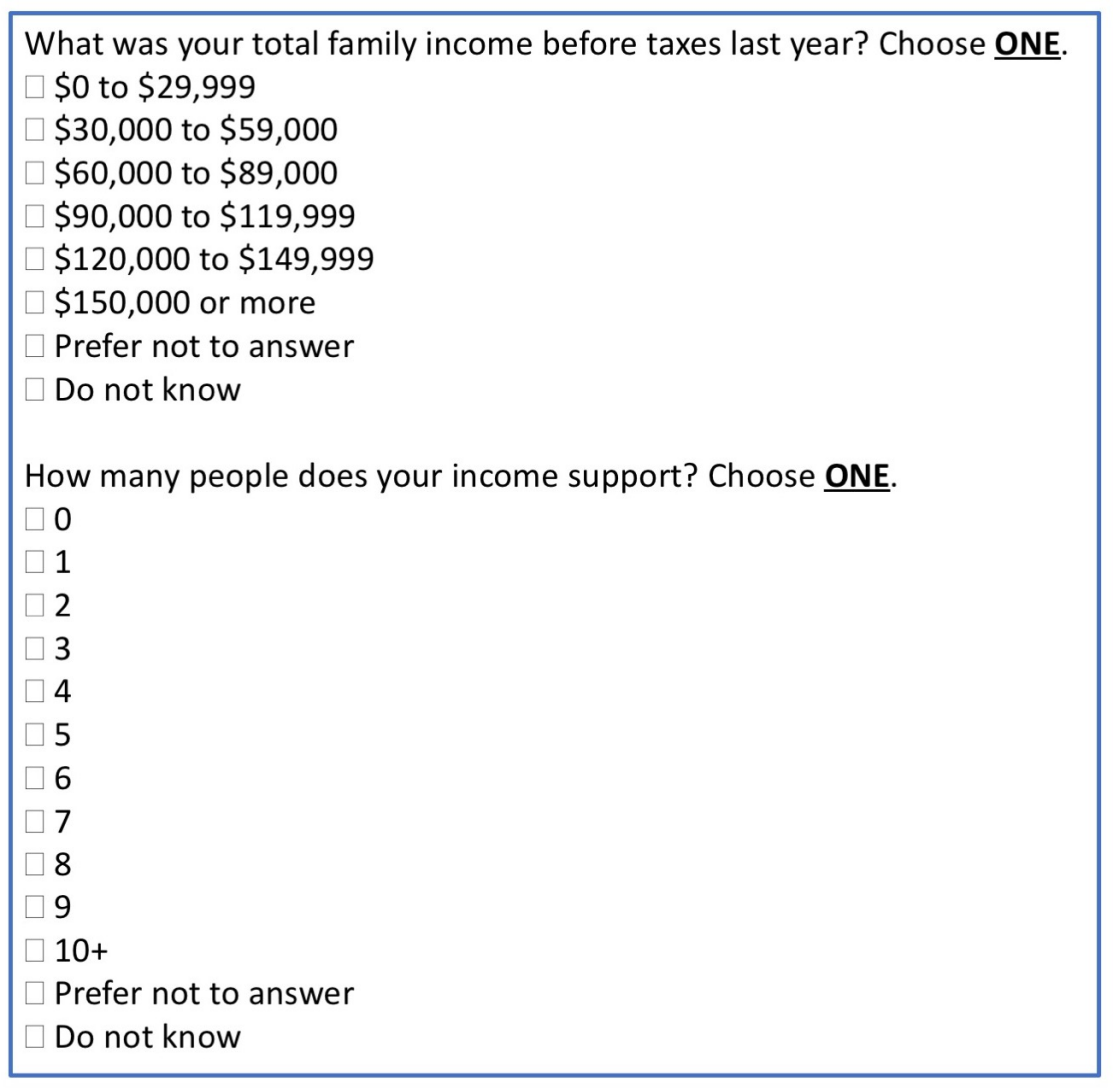
Questions about income

A sociodemographic survey was developed with the support of the regional health authority.^26^ A two-part question asked about income (Figure 1). Pilot testing occurred in 2012 in four sites.^25,26^ The St Michael’s Family Health Team began routinely administering the survey in December 2013. Clerical staff were oriented to the survey’s purpose and offered it to patients when they arrived for an appointment. Surveys were completed in the waiting room on an electronic tablet. Patients who were unable to complete the survey could have another person complete it on their behalf. In some circumstances, paper surveys were used and then the data were later manually entered by staff. The survey was only offered in English. Answers were uploaded into the patient’s electronic medical record (EMR) in a format that allowed the data to be extracted.

### Study design

A mixed-methods study was conducted. The work was supported by an advisory committee that included patients and representatives from other hospitals, the regional health authority, and the provincial health quality agency.

### Quantitative data and analysis

The study population was any patient who answered at least one question on the sociodemographic survey between 1 December 2013 and 31 March 2016. Data were used from the EMR to compare patients who completed both parts of the income question on the survey (responders) with those who selected ‘prefer not to answer‘, ‘don’t know‘, or who skipped either or both parts of the income question (non-responders). The study compared self-reported sex, preferred language, immigration status, housing type, ethnic group, and sexual orientation, using responses to the other survey questions. Neighbourhood income quintiles were assigned using the patient’s postal code and information from the 2006 Canadian census, which is a commonly used approach.^8,27,28^ Insurance claims were used to determine if a patient had a visit for a severe mental illness (for example, schizophrenia or bipolar disorder).^29^ Responders and non-responders were compared using a χ^2^ test, with the exception of age, which was analysed as a continuous variable using Mann-Whitney U test. Logistic regression modelling was used to estimate adjusted odds ratios (AOR), and prespecified a *P*-value of <0.001 as the threshold for significance. Variables were included in the regression model if they were significantly different in the univariate analysis. Because it was anticipated that language preference, immigration status, and ethnic group would be highly correlated, it was planned to include only ethnic group in the logistic regression analysis.

Participants who answered both parts of the income question were categorised into those with a household income above or below the before-tax low income cut-off (LICO), as described elsewhere.^30^ This is a common poverty measure in Canada, developed by Statistics Canada to identify households that likely spend a minimum of 20% more of their income on basic necessities than the average household.^30,31^ These results were compared with average neighbourhood income and specific billing codes submitted when patients receive government financial assistance, which provide incomes far below Canada’s LICO.

### Qualitative data and analysis

Structured interviews were conducted focused on cognitive processes and they included asking about any discomfort or difficulty answering the questions (see Supplementary Appendix S1 for the interview guide). Patients were recruited and interviewed in clinic waiting rooms, shortly after they had completed the survey, between May 2016 and July 2016. Patients recruited to be interviewed represent a convenience sample, but demographic data were collected and it was reviewed iteratively to ensure maximum variability in terms of sex and age (see Supplementary Table S1 for participant demographic information). The average length of interviews was 29 minutes. All interviews were audiorecorded and transcribed verbatim. A team of five coders analysed the transcripts using NVivo (version 11), and each transcript was analysed by ≥2 coders. A qualitative description approach was employed to analyse the transcripts.^32^ Initial codes were developed from the interview guides. A subset of transcripts was analysed and new codes arose. A codebook was developed and the remaining transcripts were analysed. Results were discussed with the entire study team to further refine the themes. The final themes were determined by consensus by the entire study team.

## Results

### Quantitative findings

During the study period, 15 221 patients were offered the survey and 14 247 (94%) answered at least one question. The median age was 44 years (interquartile range [IQR] 33–58 years). Fifty-five per cent were female, 40% were male, 1% were other (transgender, intersex, and those who selected multiple sex terms or ‘other‘), and 4% did not provide their sex. Fifty-five per cent were born in Canada, 39% were ethnic minorities, and 92% reported that English was their preferred language to speak with health providers. Three per cent reported living with severe mental illness.

**Figure 2.**
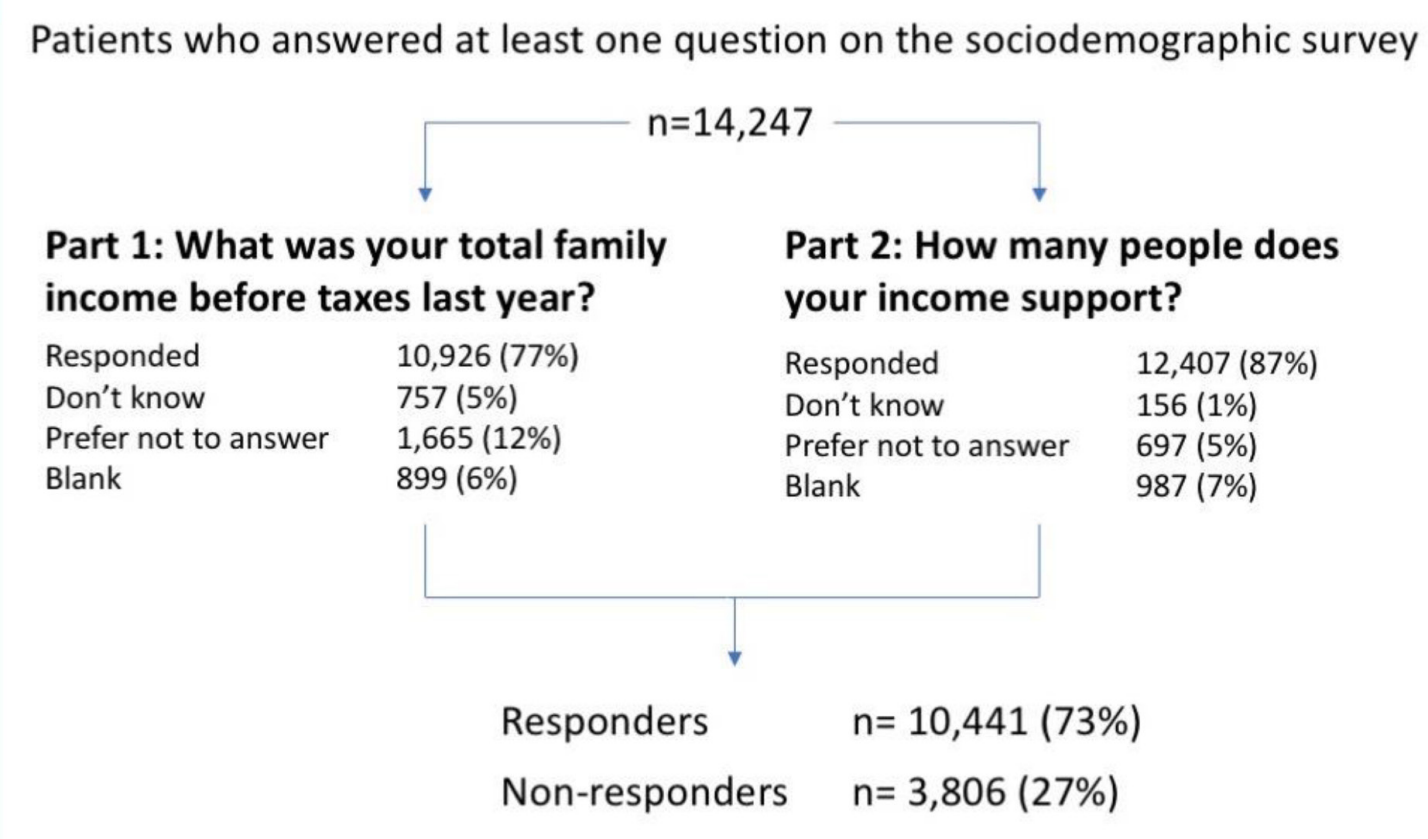
Responses to income questions. Responders are those who responded to both parts of the question. Non-responders answered ‘don’t know’, ‘prefer not to answer’, or left a question blank.

A total of 10 441 (73%) patients responded to both parts of the income question (Figure 2). In the univariate analysis, responders and non-responders differed significantly by sex, age category and median age, preferred spoken language, immigration status, housing status, ethnic group, sexual orientation, and neighbourhood income quintile (Table 1). There was no difference between the responders and non-responders with regard to a history of serious mental illness.

**Table 1.** Test of association between responding to two-part income question and sociodemographic characteristics of patients

Characteristic	**Responders,** ***n* = 10 441, *n* (%)^a^**	**Non-responders,** ***n* = 3806**, *n* (%)^a^	***P* value^b^**
**Sex**			***P*<** **0.0001**
Male	4435 (42)	1252 (33)	
Female (reference)	5738 (55)	2133 (56)	
Other^c^	98 (1)	30 (1)	
Missing	170 (2)	391 (10)	
**Age, years**			***P*<** **0.0001**
<10	99 (1)	98 (3)	
10–19	78 (1)	125 (3)	
20–29	1294 (12)	625 (16)	
30–39	2704 (26)	757 (20)	
40–49	2117 (20)	618 (16)	
50–59	1944 (19)	661 (17)	
60–69	1327 (13)	494 (13)	
≥70	878 (8)	428 (11)	
Median (IQR)	44 (34–57)	44 (31–59)	***P* =** **0.01**
**Language (spoken)**			
English (reference)	9764 (94)	3382 (89)	***P*<** **0.0001**
Other	624 (6)	316 (8)	
Missing	53 (1)	108 (3)	
**Immigration status**			***P*<** **0.0001**
Born outside Canada	4193 (40)	1689 (44)	
Canadian born (reference)	6167 (59)	1677 (44)	
Missing	81 (1)	440 (12)	
**Housing status**			***P*<** **0.0001**
Own home (reference)	4301 (41)	1173 (31)	
Rent	4846 (46)	1300 (34)	
Other	935 (9)	359 (9)	
Missing	359 (3)	974 (26)	
**Ethnic group**			***P*<** **0.0001**
White (reference)	6128 (59)	1414 (37)	
Non-White	3901 (37)	1636 (43)	
Missing	412 (4)	756 (20)	
**Sexual orientation**			***P*<** **0.0001**
Heterosexual (reference)	8173 (78)	2611 (69)	
Gay, bisexual, lesbian, queer, two-spirit, or other	1777 (17)	345 (9)	
Missing	491 (5)	850 (22)	
**Mental health**			
Severe mental illness^d^	296 (3)	107 (3)	*P* = 0.99
**Neighbourhood income quintile^e^**			
Q1 (reference)	2469 (28)	1041 (32)	***P*<** **0.0001**
Q2	1458 (17)	543 (17)	
Q3	1379 (16)	493 (15)	
Q4	1399 (16)	417 (13)	
Q5	2114 (24)	717 (22)	

^a^Unless otherwise stated. ^b^χ2 for categorical and Mann-Whitney U Test for continuous variables (age) as age data not normally distributed; bold indicates statistically significant. ^c^Includes trans-FTM (female to male), trans-MTF (male to female), intersex, ‘other‘, and those who selected multiple sex terms. ^d^Includes patients with a bill submitted that includes the diagnostic code for schizophrenia, bipolar disorder, psychosis, paranoia, or psychosis not otherwise specified. ^e^For participants with a valid postal code (*n* = 8819 responders, *n* = 3211 non-responders), linkable to 2006 Census data on income. IQR = interquartile range. Q = quintile.

The regression analysis identified that females were less likely to respond to the income question when compared with males (AOR 0.79, 95% confidence interval [CI] = 0.72 to 0.87, *P*<0.001). There was a small number of participants aged <19 years, but both those aged <29 years and those aged ≥70 years were less likely to respond than those aged 30–39 years. Non-White individuals were less likely to respond than White individuals (AOR 0.62, 95% CI = 0.56 to 0.68, *P*<0.001). LGBTQ+ individuals were more likely to respond than heterosexual individuals (AOR 1.42, 95% CI = 1.23 to 1.65, *P*<0.001) (Table 2).

**Table 2.** Adjusted odds of responding to a two-part question on income

**Covariate**	Characteristic	**AOR (CI)**	***P* value^a^**
Sex	Female	0.79 (0.72 to 0.87)	**<** **0.001**
	Other^b^	0.92 (0.54 to 1.56)	0.762
	Prefer not to answer, do not know, or missing	0.51 (0.39 to 0.66)	**<** **0.001**
	Male (reference)	1.0	n/a
Age, years	<10 versus 30–39	0.51 (0.36 to 0.74)	**<** **0.001**
	10–19 versus 30–39	0.22 (0.15 to 0.31)	**<** **0.001**
	20–29 versus 30–39	0.53 (0.46 to 0.62)	**<** **0.001**
	40–49 versus 30–39	0.97 (0.84 to 1.12)	0.659
	50–59 versus 30–39	0.84 (0.73 to 0.98)	0.022
	60–69 versus 30–39	0.78 (0.67 to 0.92)	0.003
	≥70 versus 30–39	0.66 (0.56 to 0.79)	**<** **0.001**
Housing status	Renting versus own home	1.15 (1.03 to 1.28)	0.013
	Other versus own home	0.86 (0.73 to 1.01)	0.067
	Prefer not to answer, do not know, missing versus own home	0.18 (0.15 to 0.21)	**<** **0.001**
Ethnic group	Non-White versus White	0.62 (0.56 to 0.68)	**<** **0.001**
	Prefer not to answer, do not know, or missing versus White	0.29 (0.25 to 0.34)	**<** **0.001**
Sexual orientation	Gay, bisexual, lesbian, queer, two-spirit, or other versus heterosexual	1.42 (1.23 to 1.65)	**<** **0.001**
	Prefer not to answer, do not know, or missing versus heterosexual	0.37 (0.32 to 0.43)	**<** **0.001**
Income quintile	Q2 versus Q1	1.06 (0.92 to 1.22)	0.392
	Q3 versus Q1	1.04 (0.91 to 1.2)	0.554
	Q4 versus Q1	1.14 (0.98 to 1.32)	0.091
	Q5 versus Q1	0.98 (0.86 to 1.12)	0.752

^a^Bold indicates statistically significant. ^b^Includes trans-FTM (female to male), trans-MTF (male to female), intersex, ‘other‘, and those who selected multiple sex terms. Q = quintile. n/a = not applicable.

A total of 3425 (33%) patients who responded to both parts of the income question were determined to be living below the LICO (see Supplementary Table S2). The average neighbourhood income quintile could be determined for 2920 (85%) of these individuals living below the LICO. It was found that 1291 (44%) patients with self-reported income below the LICO were living in the lowest neighbourhood income quintile and 390 (13%) were living in highest neighbourhood income quintile. In addition, 177 (20%) of the 882 individuals on government financial assistance were not identified as living below the LICO, based on their responses to the income question.

### Qualitative findings

The analysis of interviews with 27 patients identified three major themes: 1) comfort level with disclosing income; 2) knowledge of link between income and health; and 3) concerns about inaccurate representation of income. First, a range of perspectives on comfort level were found with disclosing income. Most patients did not express discomfort with answering a question about their income, when asked in the context of the primary care organisation. One patient noted:

*‘*… *I didn’t see it as intrusive or anything at all. I didn’t feel like I was giving out information that I was concerned about giving out.* *’* (Participant [P]4, male, aged 50–64 years)

A small number of patients reported concerns that the data could lead to discrimination or a loss of services in the future. One patient reported:

*‘My fear is that maybe I would lose some services or that it would affect my quality of care in one way or another.* *’* (P5, female, aged 35–49 years)

Second, the study found patients talked about their knowledge of the link between income and health. If patients understood the link between income and health they were more comfortable. One patient noted:

*‘I think your income is important … you make a hundred and fifty grand a year or more, you’re obviously going to be in better health than me*.*’* (P23, female, aged 50–64 years)

Some expressed that income data could be used to better characterise the clinic’s population and its needs. Other patients offered ideas as to how the data might be used by physicians, such as understanding patients’ stress, recognising food insecurity, and knowing which medications a patient can afford. However, in some cases, patients did not make the link between income and health, and did not understand why the question was being asked. One patient stated:

*‘… I wasn’t sure why you were asking that … when I retire, my income will go — drop dramatically. But I don’t change. So, I am not sure how that question really is valid in terms of what you’re doing.* *’* (P4, male, aged 50–64 years)

Third, it was found patients talked about concerns about the inaccurate representation of income. A number of patients found it difficult to calculate their income or did not feel that their answer truly represented their financial situation accurately. Some patients did not know their family’s income, particularly young adults living with their parents. Such patients selected ‘don’t know‘ and others attempted to guess their family’s income. Other patients thought the question did not capture a true picture of their financial situation. One patient stated that after-tax income would be more relevant than before-tax, as that is what they have available to spend. Several patients noted that their income fluctuates significantly:

*‘I had a really strange year last year where I got laid off and I got a year’s severance pay.* […] *So they gave me a really good package and so I felt like if I said* [my income was] *like over 100 000 dollars it would be very misleading.* *’* (P16, female, aged 35–49 years)

Some patients reported that they were unsure whose incomes should be included in a household income and there was confusion as to who should be included in the number of people supported by this income. This was particularly confusing for patients with less traditional family structures; for example, a parent paying child support, young adults partially supported by parents, or those sending remittances overseas. Some were not sure if non-relatives could be counted. Most patients understood the word ‘support‘, and thought it encompassed living costs such as housing, food, clothing, and education.

## Discussion

### Summary

This study analysed data from a routinely administered survey that included a two-part question on income. Approximately three-quarters of patients answered both parts of the income question, and most patients interviewed did not express discomfort. Comfort level was related to whether patients understood why the data were being collected. Neighbourhood income quintile, a commonly used proxy for an individual’s income, was not associated with responding. Patients faced difficulty in estimating their household income before taxes, particularly if incomes fluctuated significantly.

### Strengths and limitations

The strengths of this study include the pragmatic application of the sociodemographic survey into the workflow of several clinics, the large number of patients surveyed, and the use of mixed methods. There were several limitations to this study. As the study took place in a large urban centre in Canada, results are only transferrable to similar settings in the country. A limitation of the quantitative analysis is that no gold standard for income was available (for example, income tax returns), and hence it could not be evaluated whether patients answered the income question truthfully. A limitation of the qualitative component of the study is that patients were recruited in a convenience sample, when attending the clinic, and could be biased toward those who were willing to discuss their experience. A small number of the surveys were likely completed by caregivers or family members on behalf of the patient (for example, children), but data on this population were unable to be captured. The interviews did not explore why certain patients — females, ethnic minorities, and the very young and older people — were less likely to respond to the income question. Also, survey non-responders were not approached for interviews so the perspective of response bias was unable to be gained. Finally, surveys and interviews were only offered in English, and the experience of non-English speakers could certainly be different.

### Comparison with existing literature

It was surprising that more than one-fifth of patients on government financial assistance reported an income above the LICO, although this may include people who were only temporarily on social assistance or who had income from other sources. Using responses on the survey as the gold standard, it was found that average neighbourhood income quintile is not an adequate proxy. This confirms other research^33–35^ and suggests that individual-level data are preferred, particularly in mixed-income urban settings. Surveys that ask about income report non-response rates ranging from 10%–30%, depending on the setting and how the question is asked.^36,37^ In a study of 1427 hospitalised patients that used a similar sociodemographic survey, the response rate was only 54%.^38^ Other work has also found that patients find it difficult to recall their income when asked to provide a dollar value.^36,39^

### Implications for research and practice

Data on individuals’ social circumstances will be increasingly important for quality improvement efforts^13,40^ work to reduce health inequities,^41,42^ and research on health disparities.^9,43^ It was found that some patients were uncomfortable answering the question on income because of a fear of discrimination. Indeed, people with low incomes face discrimination in health care, even in the context of universal health insurance.^44^ Rather than asking patients directly to report their individual or household income, some health organisations ask about financial strain or financial need.^45–49^ A question on financial strain may also be more actionable for healthcare providers.^10^ Future research should examine the response rate and utility of asking patients about income compared with asking about financial strain, and explore how wealth, assets and debt, and other aspects of socioeconomic status modify responses. Research is also required on linking data on income or financial strain to clinic-based interventions to address poverty. At the point of care, this information could direct physicians to refer to social services, choose lower-cost treatments,^41,42,50,51^ or link to tools that identify financial benefits that improve the income of patients and families.^23^
